# Real-world effectiveness of avelumab, pembrolizumab, and enfortumab vedotin in patients with advanced urothelial carcinoma with squamous differentiation (ARON-2EV)

**DOI:** 10.1007/s00262-026-04328-9

**Published:** 2026-03-24

**Authors:** Veronica Mollica, Francesco Massari, Kazutoshi Fujita, Patrizia Giannatempo, Enrique Grande, Thomas Büttner, Maria T. Bourlon, Tarek Taha, Wataru Fukuokaya, Zin W. Myint, Renate Pichler, Kirstin Binz, Javier Molina‑Cerrillo, Jindrich Kopecky, Alfonso Gómez de Liaño, Jakub Kucharz, Ondřej Fiala, Marc R. Matrana, Ray Manneh Kopp, Mattia Alberto Di Civita, Anca Zgura, Jawaher Ansari, Randi Kihnel, Francesca De Felice, Martín Angel, Fernando Sabino M. Monteiro, Andrey Soares, Yuksel Urun, Sebastiano Buti, Daniele Santini, Matteo Santoni

**Affiliations:** 1 Medical Oncology, IRCCS Azienda Ospedaliero-Universitaria Di Bologna, Bologna, Italy; 2 Department of Medical and Surgical Sciences (DIMEC), University of Bologna, Bologna, Italy; 3 Department of Urology, Faculty of Medicine, Kindai University, Osaka, Japan; 4 Dipartimento Di Oncologia Medica, Fondazione IRCCS Istituto Nazionale Dei Tumori, Milan, Italy; 5 Department of Medical Oncology, MD Anderson Cancer Center Madrid, Madrid, Spain; 6 Department of Urology, University Hospital Bonn, Bonn, Germany; 7 Department of Hemato-Oncology, Escuela de Medicina, Instituto Nacional de Ciencias Medicas y Nutricion Salvador Zubiran, Mexico City, Mexico—Universidad Panamericana, Mexico City, Mexico; 8 Royal Marsden NHS Foundation Trust, London, UK; 9 Department of Urology, Jikei University School of Medicine, Tokyo, Japan; 10 Division of Medical Oncology, Department of Internal Medicine, Markey Cancer Center, University of Kentucky, Lexington, KY USA; 11 Department of Urology, Comprehensive Cancer Center Innsbruck, Medical University of Innsbruck, Innsbruck, Austria; 12 Division of Medical Oncology, Department of Internal Medicine, University of Kansas Cancer Center, Kansas, USA; 13 Department of Medical Oncology, Hospital Ramón y Cajal, Madrid, Spain; 14 Department of Clinical Oncology and Radiotherapy, University Hospital Hradec Kralove, Hradec Kralove, Czechia; 15 Medical Oncology Department, CHU Insular-Materno Infantil, Las Palmas, Spain; 16 Department of Uro-Oncology, Maria Sklodowska-Curie National Research Institute of Oncology Warsaw, Warsaw, Poland; 17 Department of Oncology and Radiotherapeutics, Faculty of Medicine and University Hospital in Pilsen, Charles University, Pilsen, Czech Republic; 18 Biomedical Center, Faculty of Medicine in Pilsen, Charles University, Pilsen, Czech Republic; 19 Department of Internal Medicine, Hematology/Oncology, Ochsner Medical Center, New Orleans, LA USA; 20 Clinical Oncology, Sociedad de Oncología y Hematología del Cesar, Valledupar, Colombia; 21 Department of Radiological, Oncological and Pathological Sciences, Policlinico Umberto I, University of Rome, Rome, Italy; 22 Department of Obstetrics-Radiotherapy, Alexandru Trestioreanu Institute of Oncology, “Carol Davila” University of Medicine and Pharmacy, Prof. Dr, Bucharest, Romania; 23 Medical Oncology, Tawam Hospital, Al Ain, UAE; 24 Radiation Oncology, Department of Radiological, AOU Policlinico Umberto I, Oncological and Pathological Sciences, “Sapienza” University of Rome, Rome, Italy; 25 Instituto Alexander Fleming, Ciudad De Buenos Aires, Argentina; 26 Latin American Cooperative Oncology Group—LACOG, Porto Alegre, Brazil; 27 Oncology and Hematology Department, Hospital Sírio Libanês, Brasília, Brazil; 28 Oncology Unit, Hospital Israelita Albert Einstein, São Paulo, SP Brazil; 29 Department of Medical Oncology, Faculty of Medicine, Ankara University, 06620 Ankara, Turkey; 30 Medical Oncology Unit, University Hospital of Parma, Parma, Italy; 31 Department of Medicine and Surgery, University of Parma, Parma, Italy; 32 Medical Oncology Unit, Macerata Hospital, Macerata, Italy

**Keywords:** Avelumab, Enfortumab Vedotin, Immunotherapy, NCT05290038, Pembrolizumab, Squamous differentiation, Urothelial Carcinoma

## Abstract

**Introduction:**

Avelumab, pembrolizumab, and enfortumab vedotin (EV) demonstrated efficacy in mUC following platinum-based chemotherapy. However, real-world data in patients with urothelial carcinoma with squamous differentiation (UCSD) are limited. The aim of this study is to assess the real-world clinical outcomes of avelumab, pembrolizumab, or EV in mUCSD patients.

**Materials and methods:**

The ARON-2EV study is a retrospective, international, multicenter analysis in patients with mUC treated with avelumab, pembrolizumab, or EV across 79 centers in 21 countries. Patients were divided into three cohorts: 1 (avelumab), 2 (pembrolizumab), and 3 (EV). Primary endpoints were overall survival (OS) and time on treatment (ToT). Secondary objectives included evaluating clinical factors associated with outcomes and exploring the impact of UCSD histology on response to therapy. Statistical methods included Kaplan–Meier estimates, log-rank tests, Fisher’s exact and chi-square tests, and Pearson’s correlation coefficients.

**Results:**

A total of 1918 patients, 1696 with advanced pure UC (pUC) and 222 with mUCSD (36 in cohort 1, 111 in cohort 2, and 75 in cohort 3), were included. Median OS was shorter in patients with UCSD compared to patients with pUC histology in the three cohorts (1: 13.0 vs 26.8 months, HR 2.66, *p* = 0.003; 2: 10.2 vs 18.5 months, HR 1.52, *p* = 0.008; and 3: 7.6 vs 13.1 months, HR 1.68, *p* = 0.011). Median ToT was shorter in patients with UCSD compared to patients with pUC histology in cohort 1 (3.5 vs 5.6 months, HR 1.57, *p* = 0.044) and 3 (7.6 vs 13.6 months, HR 1.83, *p* = 0.005) but not in cohort 2 (3.7 vs 4.7 months, HR 1.19, *p* = 0.177). Response to therapy was negatively correlated with UCSD histology in cohorts 2 (correlation coefficient 0.094, *p* = 0.008) and 3 (correlation coefficient 0.107, *p* = 0.021), while response to avelumab was not correlated with UCSD (correlation coefficient 0.072, *p* = 0.263).

**Conclusions:**

UCSD is a histology with a poor prognosis and response to treatments compared to pUC. Treatments activity and effectiveness in divergent differentiations should be addressed in dedicated prospective studies.

**Trial registration number:**

NCT05290038

**Supplementary Information:**

The online version contains supplementary material available at 10.1007/s00262-026-04328-9.

## Introduction

Urothelial carcinoma (UC) of the bladder and urinary tract can present different histological subtypes or divergent differentiations [1]. Recent investigations have highlighted variant histology in UC as a prognostic element in patients with locally advanced UC [2–4]. The most prevalent histological differentiation, the squamous cell feature, is identified in approximately 40% of advanced UC cases [5]. UC with squamous differentiation (UCSD) tends to be more aggressive, often associated with higher-grade concurrent UC or diagnosed at a later stage compared to pure UC (pUC) [2].

The emergence of immune checkpoint inhibitors (ICIs) has profoundly transformed the therapeutic landscape for advanced urothelial carcinoma (UC). Avelumab has been established as a standard of care maintenance treatment based on the improved overall survival (OS) and progression-free survival (PFS) over best supportive care reached in the JAVELIN Bladder 100 trial [6]. Moreover, pembrolizumab demonstrated substantial survival advantages in patients after platinum-based chemotherapy failure, as evidenced in the KEYNOTE-045 phase III clinical trial [1, 7]. A recent addition to the treatment scenario of metastatic UC is represented by the antibody–drug conjugate enfortumab vedotin (EV) that was shown to improve survival outcomes in patients previously treated with chemotherapy and immunotherapy [8].

The efficacy of immunotherapy and EV assessed in these phase III trials is based on patients with a predominant UC component, while limited data are available on the activity of these compounds on variant histologies and differentiations, including UCSD.

A recent retrospective study examining patients with UC with variant histologies or divergent differentiations treated using ICIs found that clinical response and survival were largely similar across all histological subtypes, excluding neuroendocrine types [9]. Conversely, some dated reports suggest that UCSD might hinder responsiveness to radiation and chemotherapy [10–12], but only one study reported the potential resistance of UCSD to pembrolizumab [13]. Although the mechanisms remain elusive, UCSD seems to be able to correlate with tumor progression in UC patients treated with pembrolizumab [13].

Moreover, a retrospective study evaluated clinical outcomes in a small cohort of UCSD patients treated with ICIs or EV and showed lower ORR and shorter PFS and OS in the UCSD compared to pUC [14].

Investigating ICIs and EV’s therapeutic efficacy in UCSD patients stands as a crucial effort, also in consideration of the use of combination therapy in earlier settings.

The ARON project collects a large number of Oncology Centers worldwide with the aim of reporting real-world data on genitourinary tumors. Specifically, the ARON-2 study focuses on patients with UC treated with ICIs or EV [15–18]. In this analysis, we aimed to evaluate the activity and efficacy outcomes among patients with UCSD who received treatment with avelumab, pembrolizumab, or EV in real-world clinical settings.

## Patients and methods

### Study design and patient population

This was a retrospective cohort study that analyzed clinical data from patients aged 18 years and older diagnosed with UC with pUC or UCSD and confirmed metastatic disease by radiological assessment. The study population was divided into three distinct cohorts: Cohort 1 comprised individuals who received maintenance avelumab after achieving response or stable disease with first-line platinum-based therapy; cohort 2 included patients with disease progression or recurrence following platinum-based chemotherapy and subsequently received pembrolizumab. Cohort 3 comprised patients receiving EV after progression to platinum-based chemotherapy and PD-(L)1 inhibitor. Treatments were administered between January 1, 2016, and December 31, 2024. Data were collected from 79 medical centers across 21 countries (Fig. S1).

Demographic characteristics (such as age and gender), tumor features, Eastern Cooperative Oncology Group Performance Status (ECOG-PS), metastatic site distribution, surgical history, prior chemotherapy regimens, treatment duration, and therapeutic responses were available for all participants. Treatment responses to avelumab, pembrolizumab, or EV were assessed using the Response Evaluation Criteria in Solid Tumors (RECIST), version 1.1, as evaluated locally by the treating investigator.

Clinical and pathological data were extracted from institutional medical and pathology records, following the routine protocols of each participating center. Physical examination, laboratory tests, and imaging procedures—including computed tomography and magnetic resonance imaging —were performed according to local clinical practice. Patients lacking complete clinical or outcome data were excluded from analysis.

### Study objectives

The primary objective was to investigate the outcome of patients with mUCSD receiving avelumab (cohort 1) or pembrolizumab (cohort 2) or EV (cohort 3). Thus, the study assessed overall survival (OS), time on treatment (ToT), and overall response rate (ORR). OS was defined as the interval from avelumab, pembrolizumab, or EV treatment initiation to death from any cause. ToT referred to the interval between starting avelumab, pembrolizumab, or EV therapy and treatment discontinuation for any reason, including toxicity. Tumor responses—including progressive disease (PD), stable disease (SD), partial response (PR), and complete response (CR)—were measured using RECIST version 1.1. Primary refractory disease was defined as best overall response of PD at the first radiological assessment following treatment initiation, according to RECIST version 1.1.

Time-to-event outcomes were censored at the date of last clinical or radiological assessment for patients who were alive and event-free at last follow-up. Patients lost to follow-up were censored at the date of their last documented contact.

### Statistical methods

OS between groups was compared using the Kaplan–Meier method with differences assessed via the log-rank test. Median follow-up duration was estimated through inverted Kaplan–Meier method. Cox proportional hazards models, using Schoenfeld residuals, were applied to assess the multivariable impact on patient survival, providing hazard ratios (HRs) and 95% confidence intervals (CIs). Fisher’s exact test was employed for comparing binary categorical variables, while chi-square tests were used for multiple group comparisons. Pearson’s correlation coefficient was used to assess associations between variables. A *p* value of < 0.05 was considered statistically significant for all analyses. Analyses were conducted using a complete-case approach. The proportion of missing data for each covariate was low (< 5% for all variables), and no imputation was performed.

### Ethical compliance

The study protocol was reviewed and approved by the ethics committee of the coordinating center (Marche Region, Italy; approval number 2022 39, protocol title: “ARON 2 Project”) as well as by institutional review boards at each participating site. The study adhered to the principles outlined in the Declaration of Helsinki, followed Good Clinical Practice (GCP) guidelines, and met all applicable ethical standards for biomedical research. Informed consent was obtained from all living patients; for those deceased or lost to follow-up, consent requirements were waived by the coordinating center’s review board.

## Results

### Overall study population

A total of 1918 patients, 1696 (88%) with advanced pUC and 222 (12%) with UCSD, were included in this analysis from the ARON-2EV dataset (Fig. S2). The distribution of patients with mUCSD was as follows: 36 in cohort 1 (avelumab), 111 in cohort 2 (pembrolizumab), and 75 in cohort 3 (EV) (Table 1). The rate of UCSD in cohort 1 was 14% in both patients who started avelumab therapy before or after 2020, while in cohort 2, the rate was 9% before 2020 and 11% from 2020. All patients receiving enfortumab vedotin were treated since 2020.

**Table 1:** Patients characteristics in cohort 1 (avelumab), cohort 2 (pembrolizumab), and cohort 3 (enfortumab vedotin, EV)

Characteristics	*Avelumab (Cohort 1)*				*Pembrolizumab (Cohort 2)*				*Enfortumab vedotin (Cohort 3)*			
	Overall No. (%)	UCSD No. (%)	pUC No. (%)	*p* value	Overall No. (%)	UCSD No. (%)	pUC No. (%)	*p* value	Overall No. (%)	UCSD No. (%)	pUC No. (%)	*p* value
Total patients	252 (100)	36 (100)	216 (100)	–	1155 (100)	111 (100)	1044 (100)	–	511 (100)	75 (100)	436 (100)	–
*Sex*												
Male	198 (79)	31 (86)	167 (77)	0.144	855 (74)	77 (69)	778 (75)	0.431	390 (76)	60 (80)	330 (76)	0.609
Female	54 (21)	5 (14)	49 (23)		300 (26)	34 (31)	266 (25)		121 (24)	15 (20)	106 (24)	
Age ≥ 70y	139 (55)	19 (53)	120 (56)	0.673	595 (52)	52 (47)	543 (52)	0.572	257 (50)	37 (49)	220 (50)	1.000
*Current or former smokers*												
Yes	166 (66)	24 (67)	142 (66)	1.000	735 (64)	74 (67)	661 (63)	0.657	334 (65)	54 (72)	280 (64)	0.230
No	86 (34)	12 (33)	74 (34)		420 (36)	37 (33)	383 (37)		177 (35)	21 (28)	156 (36)	
*BMI*												
≤ 25 kg/m^2^	150 (60)	26 (72)	124 (57)	**0.028**	685 (59)	75 (68)	610 (58)	0.187	300 (59)	51 (69)	249 (57)	0.107
> 25 kg/m^2^	102 (40)	10 (28)	92 (43)		470 (41)	36 (32)	434 (42)		211 (41)	24 (31)	187 (43)	
*ECOG performance status*												
0–1	229 (91)	27 (75)	202 (94)	** < 0.001**	1014 (90)	94 (85)	920 (88)	0.544	431 (84)	59 (79)	372 (85)	0.358
≥ 2	23 (9)	9 (25)	14 (6)		141 (10)	17 (15)	124 (12)		80 (16)	16 (21)	64 (15)	
*Primary tumor location*												
Upper urinary tract	72 (29)	13 (36)	59 (27)	0.223	312 (27)	25 (23)	287 (27)	0.624	137 (27)	20 (27)	117 (27)	1.000
Lower urinary tract	180 (71)	23 (64)	157 (73)		843 (73)	86 (77)	757 (73)		374 (73)	55 (73)	319 (73)	
*Metastatic disease*												
Synchronous	95 (38)	14 (39)	81 (38)	1.000	352 (30)	38 (34)	314 (30)	0.649	155 (30)	21 (28)	134 (31)	0.646
Metachronous	157 (62)	22 (61)	135 (62)		803 (70)	73 (66)	730 (70)		356 (70)	54 (72)	302 (69)	
*Common sites of metastasis*												
Lymph nodes (non-regional)	178 (71)	26 (72)	152 (70)	0.758	739 (64)	78 (70)	661 (63)	0.300	325 (64)	49 (65)	276 (63)	0.883
Lung	94 (37)	11 (31)	83 (38)	0.372	397 (34)	31 (28)	366 (35)	0.361	189 (37)	22 (29)	167 (38)	0.182
Bone	57 (23)	10 (28)	47 (22)	0.414	314 (27)	28 (25)	286 (27)	0.872	117 (23)	12 (16)	105 (24)	0.216
Liver	46 (18)	9 (25)	37 (17)	0.224	200 (17)	20 (18)	180 (17)	1.000	79 (15)	11 (15)	68 (16)	1.000
Brain	1 (1)	0 (0)	1 (1)	1.000	18 (2)	2 (2)	16 (2)	1.000	4 (1)	1 (1)	3 (1)	1.000

In cohort 1, patients with UCSD were characterized by significantly higher rates of BMI < 25 kg/m^2^ and ECOG-PS ≥ 2 (Table 1). No significant differences were found between UCSD and pUC patients in cohorts 2 and 3. The complete list of patients’ characteristics is summarized in Table 1. In bold, statistically significant data.

### Cohort 1 (Avelumab)

**Fig. 1:**
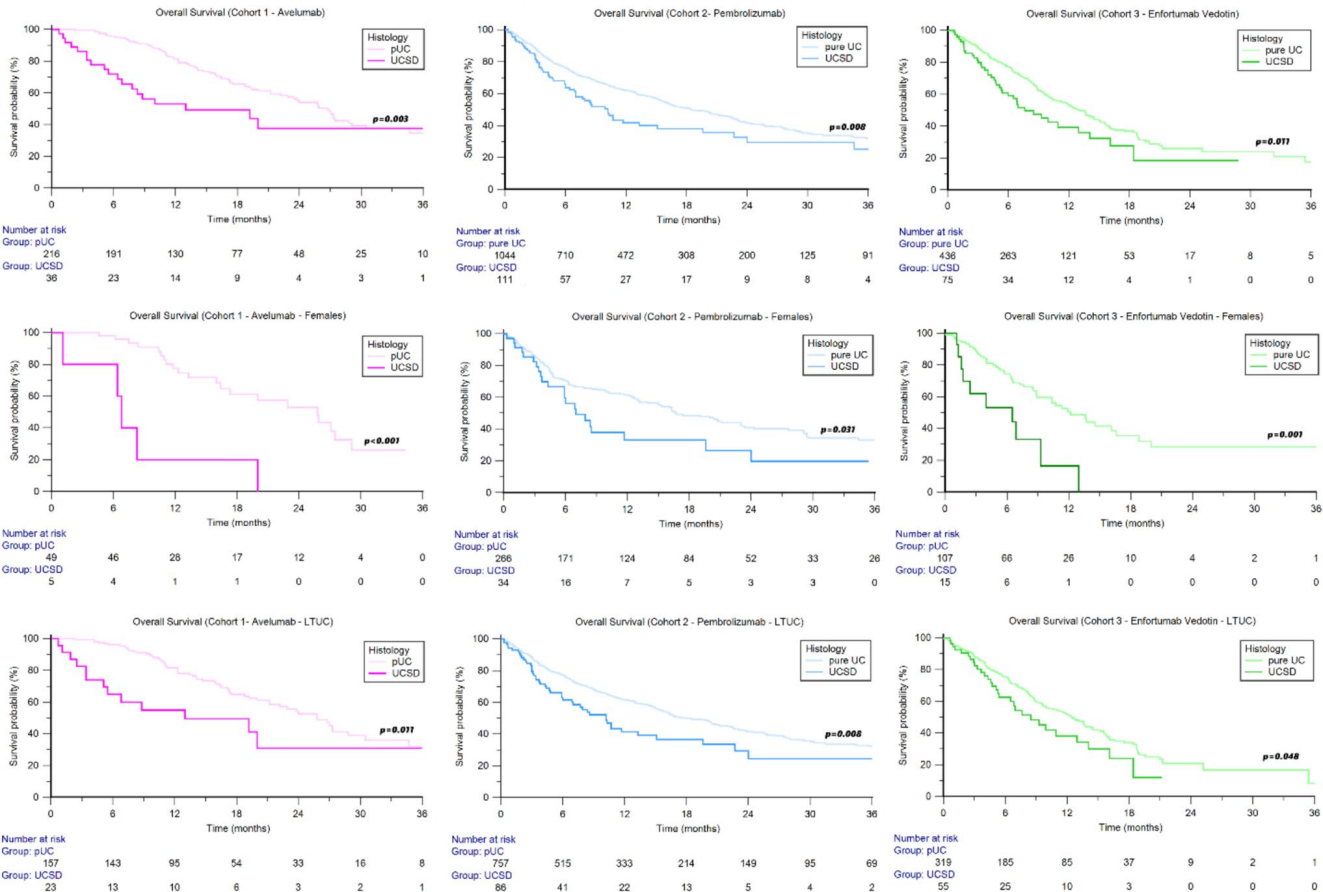
Overall survival in patients treated with avelumab (cohort 1), pembrolizumab (cohort 2), or enfortumab vedotin (cohort 3) stratified by tumor histology, gender, and site of primary tumor

In this cohort, the median follow-up was 21.8 months (95%CI 18.0–66.1). The median OS from the start of avelumab therapy was 25.8 months (95%CI 21.2–27.5) and was shorter in patients with UCSD compared to patients with pUC histology (13.0 months vs 26.8 months, HR 2.66, 95%CI 1.39–5.10, *p* = 0.003, Fig. 1), with 6-month and 12-month OS rates of 72% versus 96% and 53% versus 82%, respectively.

When stratified by sex, a numerical difference was observed in males although this did not reach statistical significance (19.2 months vs 27.0 months, HR 1.98, 95%CI 0.97–4.02, *p* = 0.058), whereas in females, the median OS was significantly lower in UCSD compared to pUC (6.8 months vs 25.6 months, HR 6.73, 95%CI 1.98–50.44, *p* < 0.001, Fig. 1).

Among patients with tumors of the lower urinary tract (LTUC), median OS was 13.0 months in UCSD and 25.8 months in pUC (HR = 2.81, 95%CI 1.34–6.32, *p* = 0.011, Fig. 1). No significant differences were found in patients with UC of the upper urinary tract (UTUC: UCSD vs pUC: NR vs 27.1 months, HR 2.42, 95%CI 0.81–7.73, *p* = 0.128).

**Fig. 2:**
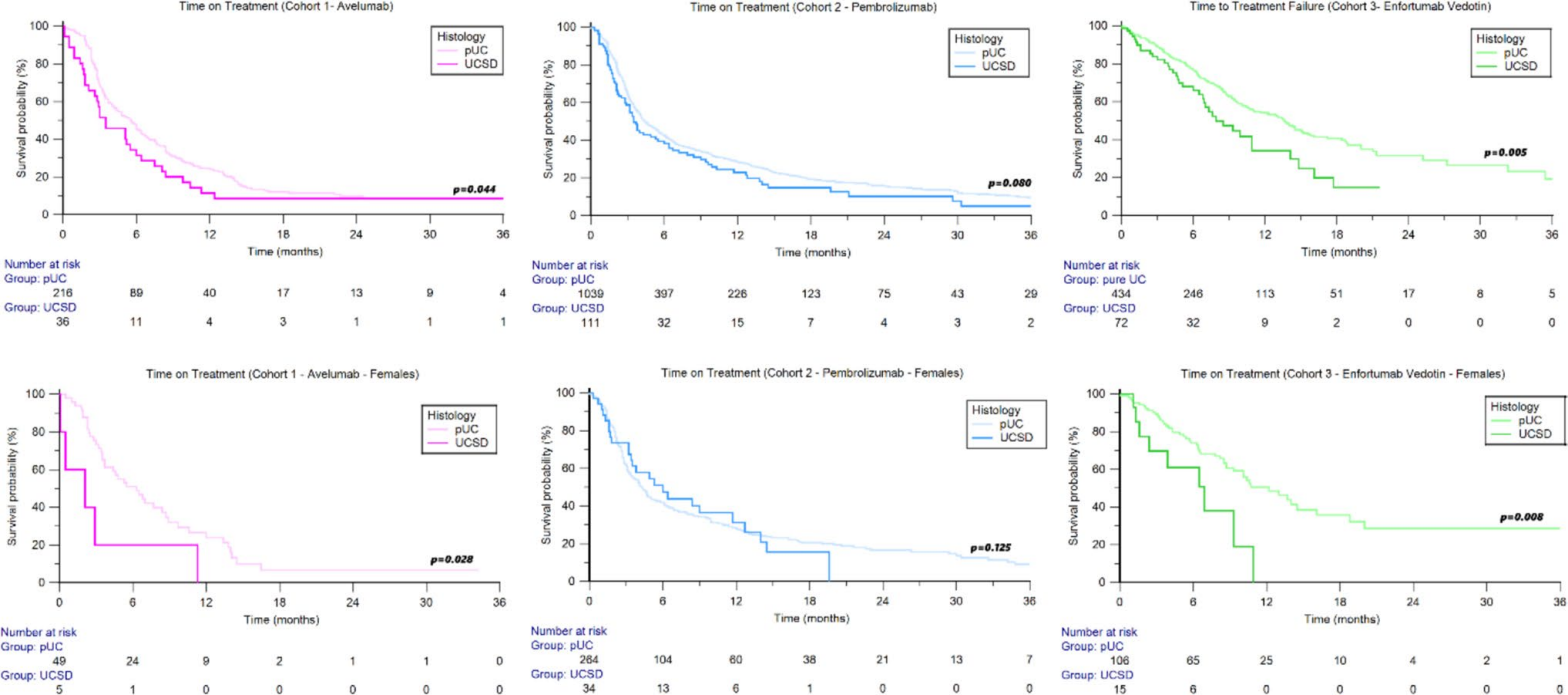
Time on treatment in patients treated with avelumab (cohort 1), pembrolizumab (cohort 2), or enfortumab vedotin (cohort 3) stratified by tumor histology

The median ToT was 5.2 months (95%CI 4.0–6.0), being shorter in patients with UCSD compared to patients with pUC histology (3.5 months vs 5.6 months, HR 1.57, *p* = 0.044, Fig. 2). Similar to OS, in male patients, no statistically significant differences in terms of ToT were observed between UCSD and pUC (5.1 months vs 5.5 months, HR 1.32, 95%CI 0.84–2.09, *p* = 0.230), while in females, the median ToT was 2.1 months in UCSD vs 6.3 months in pUC (HR 5.01, 95%CI 1.19–21.03, *p* = 0.028, Fig. 2).

The median ToT was not different in UCSD versus pUC patients in both LTUC (5.1 months vs 6.0 months, HR 1.17, 95%CI 0.71–1.93, *p* = 0.542) and UTUC subgroups (2.9 months vs 4.2 months, HR 2.64, 95%CI 0.91–6.04, *p* = 0.121).

**Fig. 3:**
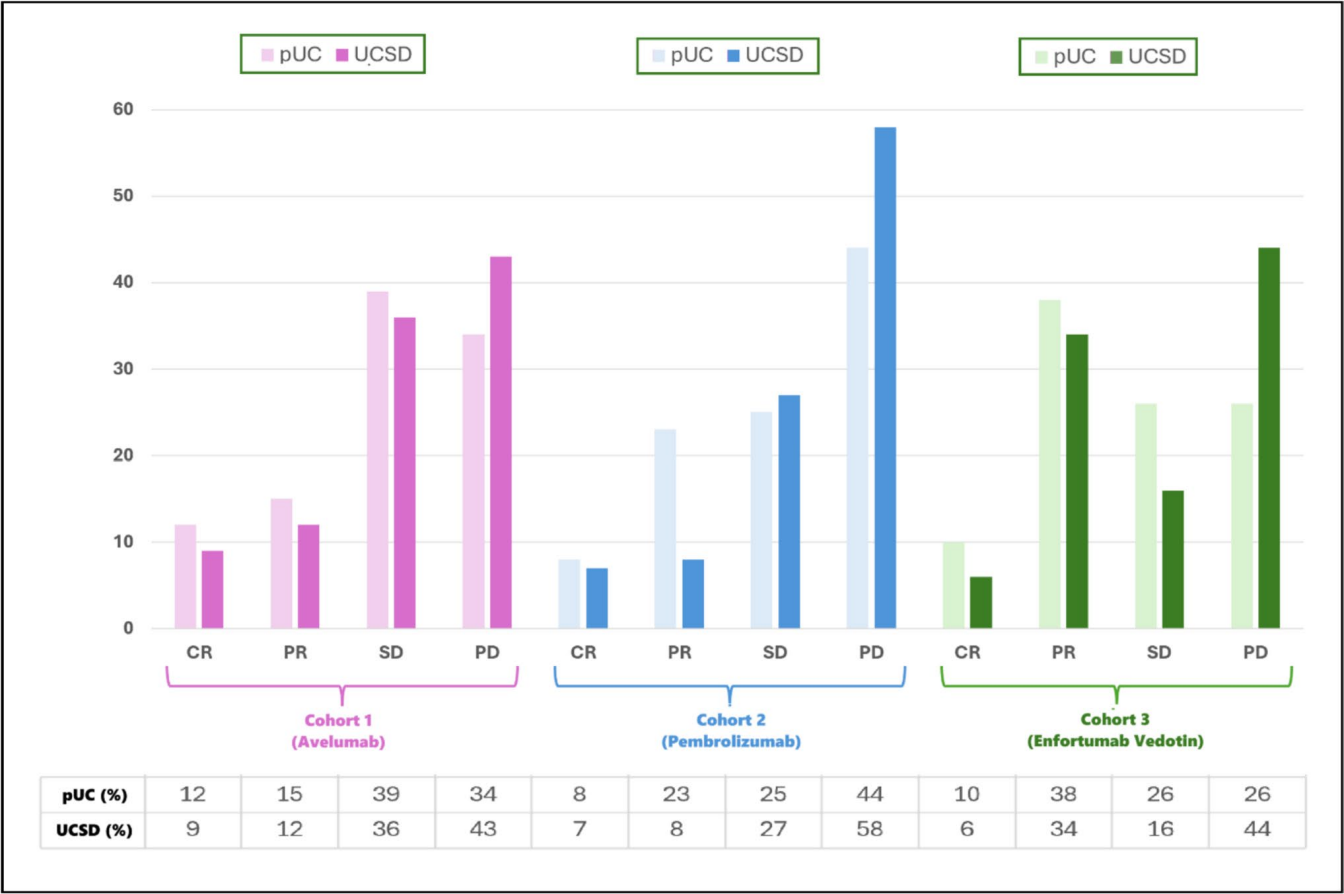
Response to therapy in pUC and UCSD patients

The ORR was 27% in pUC and 21% in UCSD (*p* = 0.408), while the rate of primary refractory disease was 34% versus 43% (*p* = 0.245), respectively (Fig. 3). Logistic regression showed a significant correlation between lymph node metastases and ORR (odds ratio 2.06, 95%CI 1.12–3.77, *p* = 0.015, Table S1) as well as between ECOG-PS ≥ 2 and primary refractory to avelumab (odds ratio 2.99, 95%CI 1.32–6.79, *p* = 0.009, Table S1).

In multivariable analyses, ECOG-PS ≥ 2 and UCSD histology were independently associated with OS (Table S2), whereas only ECOG-PS ≥ 2 was a significant predictor of ToT (Table S3).

Formal interaction tests showed a statistically significant interaction for histology × sex (Table S4).

### Cohort 2 (Pembrolizumab)

In this group, the median follow-up was 20.9 months (95% CI 19.6–22.6). The median OS was 17.9 months (95%CI 15.9–51.2). Patients with UCSD had a shorter OS compared to those with pUC (10.2 vs 18.5 months, HR 1.52, 95% CI 1.12–2.08, *p* = 0.008; Fig. 1), with 6-month and 12-month OS rates of 66% versus 76% and 42% versus 62%, respectively. Among males, median OS was 10.7 months in UCSD and 18.7 months in pUC (HR 1.38, 95%CI 0.95–2.00, *p* = 0.09). In contrast, a significant difference was observed in female patients (7.0 months vs 16.7 months, HR 1.83, 95%CI 1.06–3.16, *p* = 0.031, Fig. 1).

Patients with LTUC had a shorter median OS in the UCSD subgroup (10.2 months vs 18.5 months, HR 1.63, 95%CI 1.14–2.32, *p* = 0.008, Fig. 1), while no significant differences were found in patients with UTUC (9.9 months vs 19.0 months, HR 1.22, 95%CI 0.66–2.25, *p* = 0.535).

No statistically significant differences in terms of median ToT were found between UCSD and pUC in the overall study population (3.5 vs 4.4 months, HR 1.24, 95%CI 0.97–1.59, *p* = 0.080) and both in males (3.2 months vs 4.5 months, HR 1.39, 95%CI 0.97–1.88, *p* = 0.060) and females (3.2 months vs 4.8 months, HR 1.42, 0.94–1.93, *p* = 0.125).

The ORR was 31% in pUC and 15% in UCSD (*p* = 0.011, Fig. 3), while the rate of primary refractory was 44% versus 58%, respectively (*p* = 0.066, Fig. 3). Furthermore, UCSD was significantly associated with primary resistance to pembrolizumab (odds ratio 1.68, 95%CI 1.07–2.63, *p* = 0.023, Table S5).

In multivariable analysis for OS, ECOG-PS ≥ 2, UCSD histology, metastatic disease at diagnosis, and the presence of lung, bone, or liver metastases were associated with shorter OS (Table S6). On the other hand, ECOG-PS ≥ 2, metastasis at UC diagnosis, and the presence of metastases to distant lymph nodes, lungs, bones, or liver were associated with ToT (Table S7).

Formal interaction tests for histology×sex and histology×primary site were performed within each cohort. No statistically significant interactions were detected (Table S8).

### Cohort 3 (Enfortumab Vedotin)

In this cohort, the median follow-up was 14.5 months (95% CI 11.2–52.9). The median OS was 12.4 months (95% CI 10.4–14.1). Similar to the other two cohorts, patients with UCSD had significantly shorter OS than those with pUC (7.6 months vs 13.1 months, HR 1.68, 95%CI 1.13–2.51, *p* = 0.011, Fig. 1), with 6-month and 12-month OS rates of 59% versus 77% and 39% versus 53%, respectively. In males, median OS was 9.9 months in UCSD versus 13.7 months in pUC (HR 1.42, 95%CI 0.91–2.22, *p* = 0.122), while among females, median OS was 6.5 months in UCSD versus 12.2 months in pUC (HR 5.48 1.94–15.43, *p* = 0.001, Fig. 1).

In the LTUC subgroup, median OS was significantly shorter in patients with UCSD histology (8.5 months vs 12.4 months, HR 1.54, 95%CI 1.01–2.42, *p* = 0.048, Fig. 1). Conversely, in the UTUC subgroup, the median OS was shorter in pUC (6.0 months) than in UCSD (14.5 months, HR 2.38, 95%CI 0.98–5.70, *p* = 0.062).

Median ToT was shorter in patients with UCSD compared to patients with pUC histology (7.6 vs 13.6 months, HR 1.83, 95%CI 1.20–2.81, *p* = 0.005, Fig. 2). The difference between UCSD and pUC in terms of median ToT was statistically significant in females (6.9 months vs 12.2 months, HR 4.08 95%CI 1.45–11.48, *p* = 0.008, Fig. 2) but not in males (9.9 months vs 14.5 months, HR 1.66, 95%CI 0.97–2.69, *p* = 0.057).

The ORR was 48% in pUC and 40% in UCSD (*p* = 0.319, Fig. 3), while the rate of primary refractory was 26% versus 44% (*p* = 0.008, Fig. 3). UCSD was correlated with primary resistance to enfortumab vedotin (odds ratio 2.08, 95%CI 1.24–3.47, *p* = 0.006, Table S9).

In univariable and multivariable analyses, ECOG-PS ≥ 2 and UCSD histology were associated with both OS (Table S10) and ToT (Table S11).

Formal interaction tests showed a statistically significant interaction for histology × sex (Table S12).

## Discussion

This study provides the largest real-world cohort to date, to our knowledge, of patients with UCSD (*n* = 222) treated with modern systemic therapies, including ICIs and EV.

Across all three treatment cohorts—avelumab, pembrolizumab, and EV—patients with UCSD consistently experienced poorer outcomes than those with pUC, particularly in terms of OS and ORR. ToT was also shorter for patients with UCSD in the avelumab and EV cohorts, but not significantly different in the pembrolizumab group.

Moreover, multivariable analyses confirmed UCSD histology as an independent adverse prognostic factor for OS in all treatment settings. Interestingly, subgroup analyses further highlighted worse survival outcomes for female patients with UCSD compared to pUC (avelumab HR 6.73; pembrolizumab HR 1.83; EV HR 5.48) and for those with LTUC (avelumab HR 2.81; pembrolizumab HR 1.63; EV HR 1.54). Worse outcomes with immunotherapy in the female sex have been pointed out also in a recent meta-analysis, probably related to gender-specific immune system differences that influence immune response and escape [19]. Moreover, the female sex was found to obtain less benefit also from EV, probably due to lower nectin-4 expression in the basal histology, which is the most frequent in the female sex, and to estrogen levels impacted by smoking [20]. Regarding the worse survival outcomes in patients with LTUC, this difference could be explained by the general worse response of the squamous subtype compared to pUC, while no differences were found for UTUC patients, probably due to the intrinsic biology and molecular profile of this subgroup of patients that makes them more responsive immunotherapy. In fact, UTUCs have been reported to be enriched for MSI-high compared to LTUC [21] and to present higher tumor mutational burden [22].

These findings support the hypothesis that UCSD reflects a more aggressive disease biology in the metastatic setting, and that histologic differentiation remains clinically relevant even in the era of targeted agents and immunotherapy.

Considering the rarity of UCSD, limited data are available on this subset of patients, especially on the activity and efficacy of modern treatment strategies, such as ICIs or EV. Several previous retrospective and registry studies are in line with our results, remarking the poor prognosis and survival outcomes of patients with UCSD [23]. A single-center retrospective study evaluated treatments’ outcomes in 40 UCSD patients, of which 12 treated with EV and 38 with ICIs [14]. Similar to our results, PFS and OS were shorter for UCSD patients treated with ICIs or EV, with lower ORR to EV compared to pUC. Interestingly, in this study, patients with UCSD presented a higher prevalence of *CDKN2A*, *CDKN2B*, and *PIK3CA* alterations and lower *ERBB2* alterations in comparison with pUC, underscoring the different genomic background of this histologic divergence. Alterations in CDKN2A seem to promote a resistance to ICI in UC [24].

Another retrospective study showed similar survival outcomes as in our study in patients treated with ICIs. In particular, the study by Bakaloudi et al. included 152 patients with UCSD treated with ICIs in any line for advanced disease [25]. In UCSD patients receiving avelumab, median OS was 7 months and median PFS 3 months, while in those treated with second- or later-line ICI, median OS was 9 months and median PFS 4 months. Furthermore, a single-institution case series included 17 patients with UCSD and lower responses to ICIs in this subtype compared to pUC, but the results were not statistically significant [26].

A recent analysis of the UNITE study focused on patients with squamous differentiation (*n* = 94) compared to pUC (*n* = 366) treated with EV [27]. In this study, patients were divided into four groups: pUC, urothelial predominant (< 50% UCSD), UCSD histology predominant (50–99% UCSD), and pure squamous (100% UCSD). Median OS was 13.1 months for pUC, 12.7 for urothelial predominant, 10.6 for UCSD predominant, and 4.1 for pure UCSD patients, with the latter group being the one with worse outcomes.

Differently from our results, a retrospective analysis on 103 patients with variant histology, including 14 with squamous differentiation, treated with second-line pembrolizumab found no significant differences between the pUC and variant histology groups in terms of PFS (median 5.0 vs 10.4 months, *p* = 0.222) and OS (median 13.5 vs 23.8 months, *p* = 0.497) [28]. In line with these findings, another retrospective study on patients treated with pembrolizumab reported comparable ORR and OS among patients with squamous variant (*n* = 73) and pUC [29].

UC can be considered as a group of histologically and genomically different tumors, and each divergent histology or subtype presents relevant differences of gene expression signatures as well as of the immune microenvironment composition and, consequently, prognosis and treatment response [30]. In particular, UCSD tends to have more intra-tumoral lymphocytes, especially CD8 + [31, 32], that should actually make this subtype likely more respondent to ICIs, even though this is not translated into clinical benefit, as reported in our and other studies. The dissociation between immune microenvironment features and response raises important questions about functional T cell exhaustion, immune exclusion, or antigen presentation capacity in UCSD, among other mechanisms of resistance to ICIs. Regarding response to EV, interestingly, divergent histologies or differentiations of UC presented lower nectin-4 expression, with potential clinical implications that need to be investigated [33].

A relevant merit of our analysis is to have recollected the largest case series of patients with UCSD, to our knowledge. Nonetheless, some limitations have to be pointed out. First and foremost, the retrospective design of the study limits the strength of our results. Second, the lack of central radiologic review may lead to a misinterpretation of the tumor response. Third, the deficiency of data pertaining to the proportion of UCSD, in conjunction with the absence of a centralized pathology review, may result in an information bias, thereby necessitating that our findings be interpreted with due caution. Fourth, the use of ToT instead of progression-free survival, considering that ToT captures treatment discontinuation for any reason, including toxicity, patient preference, or logistical factors, therefore does not exclusively reflect disease control, suggesting to carefully consider this difference when interpreting ToT-based analyses. Fifth, the small number of cases in some of the analyzed subgroups (i.e., UTUC), which makes our findings as exploratory and suggests caution in interpreting *p*-values near the significance. Moreover, the study lacks molecular correlates, such as nectin-4 expression or genomic alterations. Treatment sequences and patient selection may have also introduced bias, particularly in the EV cohort. Despite these limitations, our findings provide valuable insight into the clinical behavior of UCSD in the metastatic setting.

## Conclusion

Given the rarity of the UCSD, real-world data are of great importance to try to understand treatment response and clinical outcomes of this subgroup of patients. In this large international retrospective study, UCSD in metastatic urothelial carcinoma was consistently associated with poorer outcomes across three commonly used systemic therapies: avelumab, pembrolizumab, and enfortumab vedotin. These findings highlight the prognostic significance of histologic subtypes and underscore the need for histology-informed clinical trial designs. Prospective studies incorporating molecular and histologic profiling are warranted to better define treatment strategies and improve outcomes for patients with variant urothelial carcinoma, including those with UCSD.

## Supplementary Information

Below is the link to the electronic supplementary material.

Supplementary file1 (DOCX 491 KB)

## Data Availability

No datasets were generated or analysed during the current study.

## References

[1] Netto GJ, Amin MB, Berney DM et al (2022) The 2022 World Health Organization classification of tumors of the urinary system and male genital organs-Part B: prostate and urinary tract tumors. Eur Urol 82:469–482. 10.1016/j.eururo.2022.07.002 35965208 10.1016/j.eururo.2022.07.002

[2] Minato A, Noguchi H, Moriya R et al (2021) Evaluation of the extent of variant histology in urothelial carcinoma as a predictive marker of clinical outcomes after radical cystectomy. Cancer Diagn Progn 1:345–351. 10.21873/CDP.10046 35403142 10.21873/cdp.10046 PMC8988952

[3] Minato A, Noguchi H, Tomisaki I et al (2018) Clinical significance of squamous differentiation in urothelial carcinoma of the bladder. Cancer Control. 10.1177/1073274818800269 30213195 10.1177/1073274818800269 PMC6144505

[4] Mori K, Abufaraj M, Mostafaei H et al (2020) A systematic review and meta-analysis of variant histology in urothelial carcinoma of the bladder treated with radical cystectomy. J Urol 204:1129–1140. 10.1097/JU.0000000000001305 32716694 10.1097/JU.0000000000001305

[5] Humphrey PA, Moch H, Cubilla AL, Ulbright TM, Reuter VE (2016) The 2016 WHO classification of tumours of the urinary system and male genital organs-Part B: prostate and bladder tumours. Eur Urol 70:106–119. 10.1016/j.eururo.2016.02.028 26996659 10.1016/j.eururo.2016.02.028

[6] Powles T, Park SH, Voog E et al (2020) Avelumab maintenance therapy for advanced or metastatic urothelial carcinoma. N Engl J Med 383:1218–1230. 10.1056/NEJMoa2002788 32945632 10.1056/NEJMoa2002788

[7] Bellmunt J, de Wit R, Vaughn DJ et al (2017) Pembrolizumab as second-line therapy for advanced urothelial carcinoma. N Engl J Med 376:1015–1026. 10.1056/NEJMoa1613683 28212060 10.1056/NEJMoa1613683 PMC5635424

[8] Powles T, Rosenberg JE, Sonpavde GP et al (2021) Enfortumab vedotin in previously treated advanced urothelial carcinoma. N Engl J Med 384:1125–1135. 10.1056/NEJMoa2035807 33577729 10.1056/NEJMoa2035807 PMC8450892

[9] Miller NJ, Khaki AR, Diamantopoulos LN et al (2020) Histological subtypes and response to PD-1/PD-L1 blockade in advanced urothelial cancer: a retrospective study. J Urol 204:63–69. 10.1097/JU.0000000000000761 31971495 10.1097/JU.0000000000000761 PMC7289665

[10] Akdaş A, Türkeri L (1991) The impact of squamous metaplasia in transitional cell carcinoma of the bladder. Int Urol Nephrol 23:333–336. 10.1007/BF02549603 1938228 10.1007/BF02549603

[11] Logothetis CJ, Dexeus FH, Chong C et al (1989) Cisplatin, cyclophosphamide and doxorubicin chemotherapy for unresectable urothelial tumors: the M.D. Anderson experience. J Urol 141:33–37. 10.1016/S0022-5347(17)40578-7 2908950 10.1016/s0022-5347(17)40578-7

[12] Martin JE, Jenkins BJ, Zuk RJ, Blandy JP, Baithun SI (1989) Clinical importance of squamous metaplasia in invasive transitional cell carcinoma of the bladder. J Clin Pathol 42:250–253. 10.1136/jcp.42.3.250 2703540 10.1136/jcp.42.3.250 PMC1141863

[13] Miyama Y, Morikawa T, Miyakawa J et al (2021) Squamous differentiation is a potential biomarker predicting tumor progression in patients treated with pembrolizumab for urothelial carcinoma. Pathol Res Pract 219:153364. 10.1016/j.prp.2021.153364 33610951 10.1016/j.prp.2021.153364

[14] Jindal T, Zhang L, Deshmukh P et al (2023) Impact of squamous histology on clinical outcomes and molecular profiling in metastatic urothelial carcinoma patients treated with immune checkpoint inhibitors or enfortumab vedotin. Clin Genitourin Cancer 21:e394–e404. 10.1016/j.clgc.2023.05.007 37316414 10.1016/j.clgc.2023.05.007

[15] Massari F, Santoni M, Takeshita H et al (2024) Global real-world experiences with pembrolizumab in advanced urothelial carcinoma after platinum-based chemotherapy: the ARON-2 study. Cancer Immunol Immunother 73:106. 10.1007/s00262-024-03682-w 38634928 10.1007/s00262-024-03682-w PMC11026312

[16] Rizzo A, Buti S, Giannatempo P et al (2024) Pembrolizumab in patients with advanced upper tract urothelial carcinoma: a real-world study from ARON-2 project. Clin Exp Metastasis 41:655–665. 10.1007/s10585-024-10296-0 38850317 10.1007/s10585-024-10296-0

[17] Rizzo M, Morelli F, Ürün Y et al (2025) Real-life impact of enfortumab vedotin or chemotherapy in the sequential treatment of advanced urothelial carcinoma: the ARON-2 retrospective experience. Cancer Med 14:e70479. 10.1002/cam4.70479 39980145 10.1002/cam4.70479 PMC11842279

[18] Fiala O, Massari F, Basso U et al (2024) Enfortumab vedotin following platinum chemotherapy and avelumab maintenance in patients with metastatic urothelial carcinoma: a retrospective data from the ARON-2EV study. Target Oncol 19:905–915. 10.1007/s11523-024-01099-0 39354179 10.1007/s11523-024-01099-0 PMC11557677

[19] Santoni M, Rizzo A, Mollica V et al (2022) The impact of gender on the efficacy of immune checkpoint inhibitors in cancer patients: the MOUSEION-01 study. Crit Rev Oncol Hematol 170:103596. 10.1016/j.critrevonc.2022.103596 35031442 10.1016/j.critrevonc.2022.103596

[20] Taha T, Alpert A, Baranseh J et al (2025) Sex-associated outcomes and Nectin-4 expression in Enfortumab Vedotin-treated advanced urothelial carcinoma. Ann Oncol 36:S1609

[21] Necchi A, Madison R, Pal SK et al (2021) Comprehensive genomic profiling of upper-tract and bladder urothelial carcinoma. Eur Urol Focus 7(6):1339–1346. 10.1016/j.euf.2020.08.001 32861617 10.1016/j.euf.2020.08.001

[22] Donahue TF, Bagrodia A, Audenet F et al (2018) Genomic characterization of upper-tract urothelial carcinoma in patients with lynch syndrome. JCO Precis Oncol 2:1–3. 10.1200/PO.17.00143 10.1200/PO.17.00143 PMC6404976 30854504

[23] Deuker M, Martin T, Stolzenbach F et al (2021) Bladder cancer: a comparison between non-urothelial variant histology and urothelial carcinoma across all stages and treatment modalities. Clin Genitourin Cancer 19(1):60-68.e1. 10.1016/j.clgc.2020.07.011 32782133 10.1016/j.clgc.2020.07.011

[24] Adib E, Nassar AH, Akl EW et al (2021) *CDKN2A* alterations and response to immunotherapy in solid tumors. Clin Cancer Res 27:4025–4035. 10.1158/1078-0432.CCR-21-0575 34074656 10.1158/1078-0432.CCR-21-0575 PMC8900067

[25] Bakaloudi DR, Talukder R, Enright T et al (2025) Response and survival with immune checkpoint inhibitor in patients with advanced urothelial carcinoma and histology subtypes. Clin Genitourin Cancer. 10.1016/j.clgc.2025.102356 40378559 10.1016/j.clgc.2025.102356

[26] Philip EJ, Wright F, Kim DM et al (2020) Efficacy of immune checkpoint inhibitors (ICIs) in rare histological variants of bladder cancer. J Clin Oncol 38:502. 10.1200/JCO.2020.38.6_suppl.502

[27] Jindal T, Alhalabi O, Nguyen CB et al (2024) Impact of squamous histology on outcomes with enfortumab vedotin in patients with advanced urothelial carcinoma: analysis of the UNITE study. J Clin Oncol 42:6. 10.1200/JCO.2024.42.4_suppl.6

[28] Minato A, Furubayashi N, Harada M et al (2022) Efficacy of pembrolizumab in patients with variant urothelial carcinoma: a multicenter retrospective study. Clin Genitourin Cancer 20(5):499.e1-499.e8. 10.1016/j.clgc.2022.05.001 35624001 10.1016/j.clgc.2022.05.001

[29] Kobayashi M, Narita S, Matsui Y et al (2022) Impact of histological variants on outcomes in patients with urothelial carcinoma treated with pembrolizumab: a propensity score matching analysis. BJU Int 130(2):226–234. 10.1111/bju.15510 34110696 10.1111/bju.15510

[30] Warrick JI, Sjödahl G, Kaag M et al (2019) Intratumoral heterogeneity of bladder cancer by molecular subtypes and histologic variants. Eur Urol 75:18–22. 10.1016/j.eururo.2018.09.003 30266310 10.1016/j.eururo.2018.09.003

[31] Li H, Zhang Q, Shuman L et al (2020) Evaluation of PD-L1 and other immune markers in bladder urothelial carcinoma stratified by histologic variants and molecular subtypes. Sci Rep 10:1439. 10.1038/s41598-020-58351-6 31996725 10.1038/s41598-020-58351-6 PMC6989654

[32] Jung M, Rose M, Knuechel R et al (2023) Characterisation of tumour-immune phenotypes and PD-L1 positivity in squamous bladder cancer. BMC Cancer 23:113. 10.1186/s12885-023-10576-0 36726072 10.1186/s12885-023-10576-0 PMC9890720

[33] Hoffman-Censits JH, Lombardo KA, Parimi V et al (2021) Expression of Nectin-4 in bladder urothelial carcinoma, in morphologic variants, and nonurothelial histotypes. Appl Immunohistochem Mol Morphol 29:619–625. 10.1097/PAI.0000000000000938 33901032 10.1097/PAI.0000000000000938 PMC8429050

