# Supplementary material for: Real-world effectiveness of avelumab, pembrolizumab, and enfortumab vedotin in patients with advanced urothelial carcinoma with squamous differentiation (ARON-2EV)
Source: Cancer Immunol Immunother. 2026 Mar 24;75(4):119. doi: 10.1007/s00262-026-04328-9 (PMC13013894; doi:10.1007/s00262-026-04328-9)
Supplement: Supplementary file 1 — Supplementary file1 (DOCX 491 KB) [file 262_2026_4328_MOESM1_ESM.docx]

**Supplementary Materials**

**Table S1.** Responders, primary refractory and relative Odds Ratios in Cohort 1.

|  | ***Number of responders*** | ***ORR*** | ***Odds***  ***Ratio (95%CI)*** | ***P -value*** | ***Number of primary refractory patients*** | ***Rate of primary refractory*** | ***Odds Ratio (95%CI)*** | ***P -value*** |
| --- | --- | --- | --- | --- | --- | --- | --- | --- |
| ***UCSD vs pUC*** | 8 vs 58 | 21% vs  27% | 0.86 (0.39-1.87) | 0.694 | 15 vs 73 | 43% vs  34% | 1.39 (0.67-2.87) | 0.386 |
| ***ECOG-PS≥2 vs 0-1*** | 3 vs 63 | 8% vs 29% | 0.41 (0.14-1.24) | 0.086 | 13 vs 75 | 57% vs  33% | 2.99 (1.32-6.79) | **0.009** |
| ***Metastatic disease at diagnosis (Yes vs No)*** | 26 vs 40 | 27% vs 25% | 1.09 (0.65-1.83) | 0.757 | 33 vs 55 | 35% vs  35% | 1.02 (0.62-1.68) | 0.940 |
| ***Lymph node metastases (Yes vs No)*** | 53 vs 13 | 30% vs 18% | 2.06 (1.12-3.77) | **0.015** | 56 vs 32 | 31% vs  43% | 0.62 (0.37-1.04) | 0.072 |
| ***Lung metastases***  ***(Yes vs No)*** | 21 vs 45 | 22% vs 28% | 0.76 (0.45-1.31) | 0.322 | 33 vs 55 | 35% vs  35% | 1.12 (0.67-1.85) | 0.673 |
| ***Bone metastases***  ***(Yes vs No)*** | 14 vs 52 | 25% vs 27% | 0.84 (0.46-1.54) | 0.568 | 19 vs 69 | 33% vs  35% | 0.84 (0.47-1.52) | 0.576 |
| ***Liver metastases***  ***(Yes vs No)*** | 12 vs 54 | 26% vs 26% | 1.13 (0.59-2.18) | 0.710 | 17 vs 71 | 37% vs  34% | 1.21 (0.65-2.27) | 0.554 |

**Table S2.** Univariable and Multivariable analyses in patients treated with Avelumab (Cohort 1).

| **Overall Survival (Overall population)** | **Univariable Cox Regression** | | **Multivariable Cox Regression** | |
| --- | --- | --- | --- | --- |
|  | **HR (95%CI)** | ***p-value*** | **HR (95%CI)** | ***p-value*** |
| Sex (females vs males) | 1.44 (0.96−2.15) | 0.081 |  |  |
| Age ≥70y (Y vs N) | 0.92 (0.64−1.32) | 0.643 |  |  |
| ECOG PS (≥ 2 vs 0-1) | 6.95 (4.34−11.13) | **<0.001** | 5.54 (3.25−9.46) | **<0.001** |
| Histology (UCSD vs pUC) | 2.08 (1.26−3.43) | **0.004** | 1.66 (1.01−2.76) | **0.045** |
| Primary site (bladder vs UTUC) | 1.10 (0.75−1.62) | 0.635 |  |  |
| Metastatic at diagnosis (Y vs N) | 1.37 (0.95−1.97) | 0.092 |  |  |
| Lymph node (Y vs N) | 0.87 (0.60−1.28) | 0.487 |  |  |
| Lung metastases (Y vs N) | 1.20 (0.84−1.73) | 0.318 |  |  |
| Bone metastases (Y vs N) | 1.44 (0.96−2.15) | 0.075 |  |  |
| Liver metastases (Y vs N) | 2.07 (1.32−3.23) | **0.001** | 1.64 (0.99-2.70) | 0.055 |
| ECOG-PS = Eastern Cooperative Oncology Group-Performance Status; pUC = pure Urothelial Carcinoma; UCSD = Urothelial Carcinoma with squamous differentiation; UTUC = Upper Tract Urothelial Carcinoma; Y = yes; N = no | | | | |

**Table S3.** Univariable and Multivariable analyses in patients treated with Avelumab (Cohort 1).

| **Time on Treatment (Overall population)** | **Univariable Cox Regression** | | **Multivariable Cox Regression** | |
| --- | --- | --- | --- | --- |
|  | **HR (95%CI)** | ***p-value*** | **HR (95%CI)** | ***p-value*** |
| Sex (females vs males) | 1.04 (0.77−1.42) | 0.795 |  |  |
| Age ≥70y (Y vs N) | 0.97 (0.75−1.24) | 0.787 |  |  |
| ECOG PS (≥ 2 vs 0-1) | 2.84 (1.88−4.31) | **<0.001** | - | **-** |
| Histology (UCSD vs pUC) | 1.40 (0.96−2.04) | 0.083 |  |  |
| Primary site (bladder vs UTUC) | 1.27 (0.96−1.67) | 0.096 |  |  |
| Metastatic at diagnosis (Y vs N) | 1.10 (0.85−1.42) | 0.482 |  |  |
| Lymph node (Y vs N) | 0.77 (0.59−1.01) | 0.061 |  |  |
| Lung metastases (Y vs N) | 1.06 (0.81−1.38) | 0.662 |  |  |
| Bone metastases (Y vs N) | 0.88 (0.65−1.20) | 0.424 |  |  |
| Liver metastases (Y vs N) | 1.34 (0.97−1.86) | 0.080 |  |  |
| ECOG-PS = Eastern Cooperative Oncology Group-Performance Status; pUC = pure Urothelial Carcinoma; UCSD = Urothelial Carcinoma with squamous differentiation; UTUC = Upper Tract Urothelial Carcinoma; Y = yes; N = no | | | | |

**Table S4.** Formal interaction tests for histology×sex and histology×primary site in patients treated with Avelumab (Cohort 1).

| **Overall Survival (Overall population)** | **Univariable Cox Regression** | | **Multivariable Cox Regression** | |
| --- | --- | --- | --- | --- |
|  | **HR (95%CI)** | ***p-value*** | **HR (95%CI)** | ***p-value*** |
| Histology×sex | 2.02 (1.37−2.98) | **<0.001** | 2.82 (1.33−5.98) | **0.007** |
| Histology×primary site | 1.51 (1.08−2.10) | **0.015** | 0.71 (0.35−1.45) | 0.346 |
| **Time on Treatment (Overall population)** | **Univariable Cox Regression** | | **Multivariable Cox Regression** | |
|  | **HR (95%CI)** | ***p-value*** | **HR (95%CI)** | ***p-value*** |
| Histology×sex | 1.40 (1.02−1.92) | **0.036** | 0.97 (0.47−2.04) | 0.946 |
| Histology×primary site | 1.39 (1.07−1.81) | **0.015** | 1.41 (0.77−2.58) | 0.260 |

**Table S5.** Responders, primary refractory and relative Odds Ratios in Cohort 2.

|  | ***Number of responders*** | ***ORR*** | ***Odds***  ***Ratio (95%CI)*** | ***P -value*** | ***Number of primary refractory patients*** | ***Rate of primary refractory*** | ***Odds Ratio (95%CI)*** | ***P -value*** |
| --- | --- | --- | --- | --- | --- | --- | --- | --- |
| ***UCSD vs pUC*** | 17 vs 324 | 15% vs  31% | 0.81 (0.25-2.64) | 0.727 | 64 vs 459 | 58% vs  44% | 1.68 (1.07-2.63) | **0.023** |
| ***ECOG-PS≥2 vs 0-1*** | 20 vs 321 | 14% vs 32% | 0.34 (0.20-0.58) | **<0.001** | 96 vs 427 | 68% vs  42% | 2.21 (1.53-3.20) | **<0.001** |
| ***Metastatic disease at diagnosis***  ***(Yes vs No)*** | 88 vs 253 | 25% vs 32% | 0.64 (0.47-0.87) | **0.004** | 199 vs 324 | 57% vs  40% | 1.48 (1.14-1.93) | **0.003** |
| ***Lymph node metastases***  ***(Yes vs No)*** | 240 vs 101 | 32% vs 24% | 1.35 (1.00-1.82) | **0.045** | 344 vs 179 | 47% vs  43% | 1.01 (0.78-1.32) | 0.919 |
| ***Lung metastases***  ***(Yes vs No)*** | 122 vs 219 | 31% vs 29% | 1.14 (0.85-1.52) | 0.389 | 181 vs 342 | 46% vs  45% | 1.09 (0.84-1.42) | 0.517 |
| ***Bone metastases***  ***(Yes vs No)*** | 64 vs 277 | 20% vs 33% | 0.50 (0.35-0.70) | **<0.001** | 170 vs 353 | 54% vs  42% | 1.39 (1.06-1.82) | **0.018** |
| ***Liver metastases***  ***(Yes vs No)*** | 42 vs 299 | 20% vs 32% | 0.61 (0.41-0.91) | **0.012** | 121 vs 402 | 61% vs  42% | 1.78 (1.30-2.45) | **<0.001** |

**Table S6.** Univariable and Multivariable analyses in patients receiving Pembrolizumab (Cohort 2).

| **Overall Survival (Overall population)** | **Univariable Cox Regression** | | **Multivariable Cox Regression** | |
| --- | --- | --- | --- | --- |
|  | **HR (95%CI)** | ***p-value*** | **HR (95%CI)** | ***p-value*** |
| Sex (females vs males) | 1.12 (0.95−1.32) | 0.173 |  |  |
| Age ≥70y (Y vs N) | 1.08 (0.93−1.26) | 0.286 |  |  |
| ECOG PS (≥ 2 vs 0-1) | 2.77 (2.24−3.41) | **<0.001** | 2.36 (1.88−2.96) | **<0.001** |
| Histology (UCSD vs pUC) | 1.43 (1.09−1.87) | **0.008** | 1.37 (1.05−1.80) | **0.020** |
| Primary site (bladder vs UTUC) | 0.99 (0.83−1.17) | 0.869 |  |  |
| Metastatic at diagnosis (Y vs N) | 1.27 (1.09−1.48) | **0.002** | 1.21 (1.03−1.44) | **0.024** |
| Lymph node (Y vs N) | 0.96 (0.82−1.12) | 0.593 |  |  |
| Lung metastases (Y vs N) | 1.27 (1.09−1.47) | **0.003** | 1.19 (1.01−1.41) | **0.041** |
| Bone metastases (Y vs N) | 1.52 (1.30−1.78) | **<0.001** | 1.36 (1.14−1.62) | **0.002** |
| Liver metastases (Y vs N) | 1.75 (1.46−2.09) | **<0.001** | 1.60 (1.31-1.95) | **<0.001** |
| ECOG-PS = Eastern Cooperative Oncology Group-Performance Status; pUC = pure Urothelial Carcinoma; UCSD = Urothelial Carcinoma with squamous differentiation; UTUC = Upper Tract Urothelial Carcinoma; Y = yes; N = no | | | | |

**Table S7.** Univariable and Multivariable analyses in patients receiving Pembrolizumab (Cohort 2).

| **Time on Treatment (Overall population)** | **Univariable Cox Regression** | | **Multivariable Cox Regression** | |
| --- | --- | --- | --- | --- |
|  | **HR (95%CI)** | ***p-value*** | **HR (95%CI)** | ***p-value*** |
| Sex (females vs males) | 0.99 (0.87−1.14) | 0.927 |  |  |
| Age ≥70y (Y vs N) | 0.99 (0.87−1.11) | 0.811 |  |  |
| ECOG PS (≥ 2 vs 0-1) | 1.60 (1.34−1.93) | **<0.001** | 1.50 (1.24−1.80) | **<0.001** |
| Histology (UCSD vs pUC) | 1.22 (0.97−1.52) | 0.083 |  |  |
| Primary site (bladder vs UTUC) | 1.10 (0.96−1.26) | 0.153 |  |  |
| Metastatic at diagnosis (Y vs N) | 1.20 (1.06−1.37) | **0.005** | 1.16 (1.02−1.32) | **0.026** |
| Lymph node (Y vs N) | 0.88 (0.77−0.99) | **0.041** | 0.91 (0.80−1.04) | 0.158 |
| Lung metastases (Y vs N) | 1.19 (1.05−1.35) | **0.007** | 1.12 (0.98−1.27) | 0.092 |
| Bone metastases (Y vs N) | 1.28 (1.12−1.46) | **<0.001** | 1.21 (1.05−1.38) | **0.006** |
| Liver metastases (Y vs N) | 1.42 (1.22−1.66) | **<0.001** | 1.37 (1.17-1.59) | **<0.001** |
| ECOG-PS = Eastern Cooperative Oncology Group-Performance Status; pUC = pure Urothelial Carcinoma; UCSD = Urothelial Carcinoma with squamous differentiation; UTUC = Upper Tract Urothelial Carcinoma; Y = yes; N = no | | | | |

**Table S8.** Formal interaction tests for histology×sex and histology×primary site in patients treated with Pembrolizumab (Cohort 2).

| **Overall Survival (Overall population)** | **Univariable Cox Regression** | | **Multivariable Cox Regression** | |
| --- | --- | --- | --- | --- |
|  | **HR (95%CI)** | ***p-value*** | **HR (95%CI)** | ***p-value*** |
| Histology×sex | 1.31 (1.09−1.57) | **0.004** | 1.48 (0.99−2.20) | 0.051 |
| Histology×primary site | 1.24 (1.02−1.51) | **0.031** | 0.86 (0.55−1.34) | 0.500 |
| **Time on Treatment (Overall population)** | **Univariable Cox Regression** | | **Multivariable Cox Regression** | |
|  | **HR (95%CI)** | ***p-value*** | **HR (95%CI)** | ***p-value*** |
| Histology×sex | 1.09 (0.93−1.27) | 0.300 |  |  |
| Histology×primary site | 1.11 (0.94−1.32) | 0.208 |  |  |
| pUC = pure Urothelial Carcinoma; UCSD = Urothelial Carcinoma with squamous differentiation; UTUC = Upper Tract Urothelial Carcinoma | | | | |

**Table S9.** Responders, primary refractory and relative Odds Ratios in Cohort 3.

|  | ***Number of responders*** | ***ORR*** | ***Odds***  ***Ratio (95%CI)*** | ***P -value*** | ***Number of primary refractory patients*** | ***Rate of primary refractory*** | ***Odds Ratio (95%CI)*** | ***P -value*** |
| --- | --- | --- | --- | --- | --- | --- | --- | --- |
| ***UCSD vs pUC*** | 30 vs 209 | 40% vs  48% | 0.73 (0.43-1.24) | 0.242 | 33 vs 113 | 44% vs  26% | 2.08 (1.24-3.47) | **0.006** |
| ***ECOG-PS≥2 vs 0-1*** | 31 vs 208 | 39% vs 48% | 0.56 (0.36-0.87) | **0.009** | 41 vs 105 | 51% vs  24% | 2.36 (1.54-3.61) | **<0.001** |
| ***Metastatic disease at diagnosis***  ***(Yes vs No)*** | 81 vs 158 | 52% vs 44% | 1.18 (0.83-1.68) | 0.351 | 48 vs 98 | 30% vs  28% | 1.02 (0.70-1.49) | 0.919 |
| ***Lymph node metastases***  ***(Yes vs No)*** | 170 vs 69 | 52% vs 37% | 1.59 (1.11-2.27) | **0.010** | 87 vs 59 | 27% vs  32% | 0.68 (0.47-0.99) | **0.047** |
| ***Lung metastases***  ***(Yes vs No)*** | 78 vs 161 | 41% vs 50% | 0.65 (0.46-0.93) | **0.017** | 67 vs 79 | 35% vs  25% | 1.54 (1.07-2.23) | **0.022** |
| ***Bone metastases***  ***(Yes vs No)*** | 56 vs 183 | 48% vs 46% | 0.86 (0.59-1.27) | 0.459 | 37 vs 109 | 32% vs  28% | 1.06 (0.70-1.62) | 0.771 |
| ***Liver metastases***  ***(Yes vs No)*** | 37 vs 202 | 47% vs 47% | 0.94 (0.59-1.49) | 0.796 | 30 vs 116 | 38% vs  27% | 1.59 (0.99-2.55) | 0.057 |

**Table S10.** Univariable and Multivariable analyses in patients treated with Enfortumab Vedotin (Cohort 3).

| **Overall Survival (Overall population)** | **Univariable Cox Regression** | | **Multivariable Cox Regression** | |
| --- | --- | --- | --- | --- |
|  | **HR (95%CI)** | ***p-value*** | **HR (95%CI)** | ***p-value*** |
| Sex (females vs males) | 1.12 (0.86−1.46) | 0.384 |  |  |
| Age ≥70y (Y vs N) | 0.91 (0.73−1.15) | 0.434 |  |  |
| ECOG PS (≥ 2 vs 0-1) | 2.30 (1.76−3.02) | **<0.001** | 2.42 (1.77−3.32) | **<0.001** |
| Histology (UCSD vs pUC) | 1.55 (1.10−2.18) | **0.011** | 1.62 (1.15−2.30) | **0.006** |
| Primary site (bladder vs UTUC) | 1.22 (0.94−1.58) | 0.136 |  |  |
| Metastatic at diagnosis (Y vs N) | 1.05 (0.83−1.33) | 0.700 |  |  |
| Lymph node (Y vs N) | 1.01 (0.79−1.30) | 0.906 |  |  |
| Lung metastases (Y vs N) | 1.40 (1.11−1.77) | **0.005** | 1.29 (0.99−1.67) | 0.055 |
| Bone metastases (Y vs N) | 1.07 (0.82−1.40) | 0.617 |  |  |
| Liver metastases (Y vs N) | 1.67 (1.24−2.25) | **<0.001** | 1.28 (0.91-1.80) | 0.156 |
| ECOG-PS = Eastern Cooperative Oncology Group-Performance Status; pUC = pure Urothelial Carcinoma; UCSD = Urothelial Carcinoma with squamous differentiation; UTUC = Upper Tract Urothelial Carcinoma; Y = yes; N = no | | | | |

**Table S11.** Univariable and Multivariable analyses in patients treated with Enfortumab Vedotin (Cohort 3).

| **Time on Treatment (Overall population)** | **Univariable Cox Regression** | | **Multivariable Cox Regression** | |
| --- | --- | --- | --- | --- |
|  | **HR (95%CI)** | ***p-value*** | **HR (95%CI)** | ***p-value*** |
| Sex (females vs males) | 1.17 (0.89−1.52) | 0.261 |  |  |
| Age ≥70y (Y vs N) | 1.04 (0.82−1.31) | 0.763 |  |  |
| ECOG PS (≥ 2 vs 0-1) | 1.75 (1.31−2.34) | **<0.001** | 2.02 (1.44−2.84) | **0.001** |
| Histology (UCSD vs pUC) | 1.65 (1.16−2.35) | **0.006** | 1.65 (1.16−2.37) | **0.006** |
| Primary site (bladder vs UTUC) | 1.12 (0.86−1.46) | 0.402 |  |  |
| Metastatic at diagnosis (Y vs N) | 1.04 (0.81−1.33) | 0.787 |  |  |
| Lymph node (Y vs N) | 1.02 (0.79−1.31) | 0.883 |  |  |
| Lung metastases (Y vs N) | 1.11 (0.87−1.42) | 0.404 |  |  |
| Bone metastases (Y vs N) | 0.97 (0.74−1.28) | 0.856 |  |  |
| Liver metastases (Y vs N) | 1.57 (1.15−2.14) | **0.005** | 1.27 (0.89-1.82) | 0.187 |
| ECOG-PS = Eastern Cooperative Oncology Group-Performance Status; pUC = pure Urothelial Carcinoma; UCSD = Urothelial Carcinoma with squamous differentiation; UTUC = Upper Tract Urothelial Carcinoma; Y = yes; N = no | | | | |

**Table S12.** Formal interaction tests for histology×sex and histology×primary site in patients treated with Pembrolizumab (Cohort 2).

| **Overall Survival (Overall population)** | **Univariable Cox Regression** | | **Multivariable Cox Regression** | |
| --- | --- | --- | --- | --- |
|  | **HR (95%CI)** | ***p-value*** | **HR (95%CI)** | ***p-value*** |
| Histology×sex | 1.63 (1.26−2.12) | **<0.001** | 1.93 (1.27−2.93) | 0.002 |
| Histology×primary site | 1.56 (1.07−2.28) | **0.022** | 0.75 (0.40−1.38) | 0.352 |
| **Time on Treatment (Overall population)** | **Univariable Cox Regression** | | **Multivariable Cox Regression** | |
|  | **HR (95%CI)** | ***p-value*** | **HR (95%CI)** | ***p-value*** |
| Histology×sex | 1.63 (1.25−2.14) | **<0.001** |  |  |
| Histology×primary site | 1.49 (0.99−2.23) | 0.053 |  |  |

**Figure S1.** Map of Countries participating to the ARON-2EV Study.


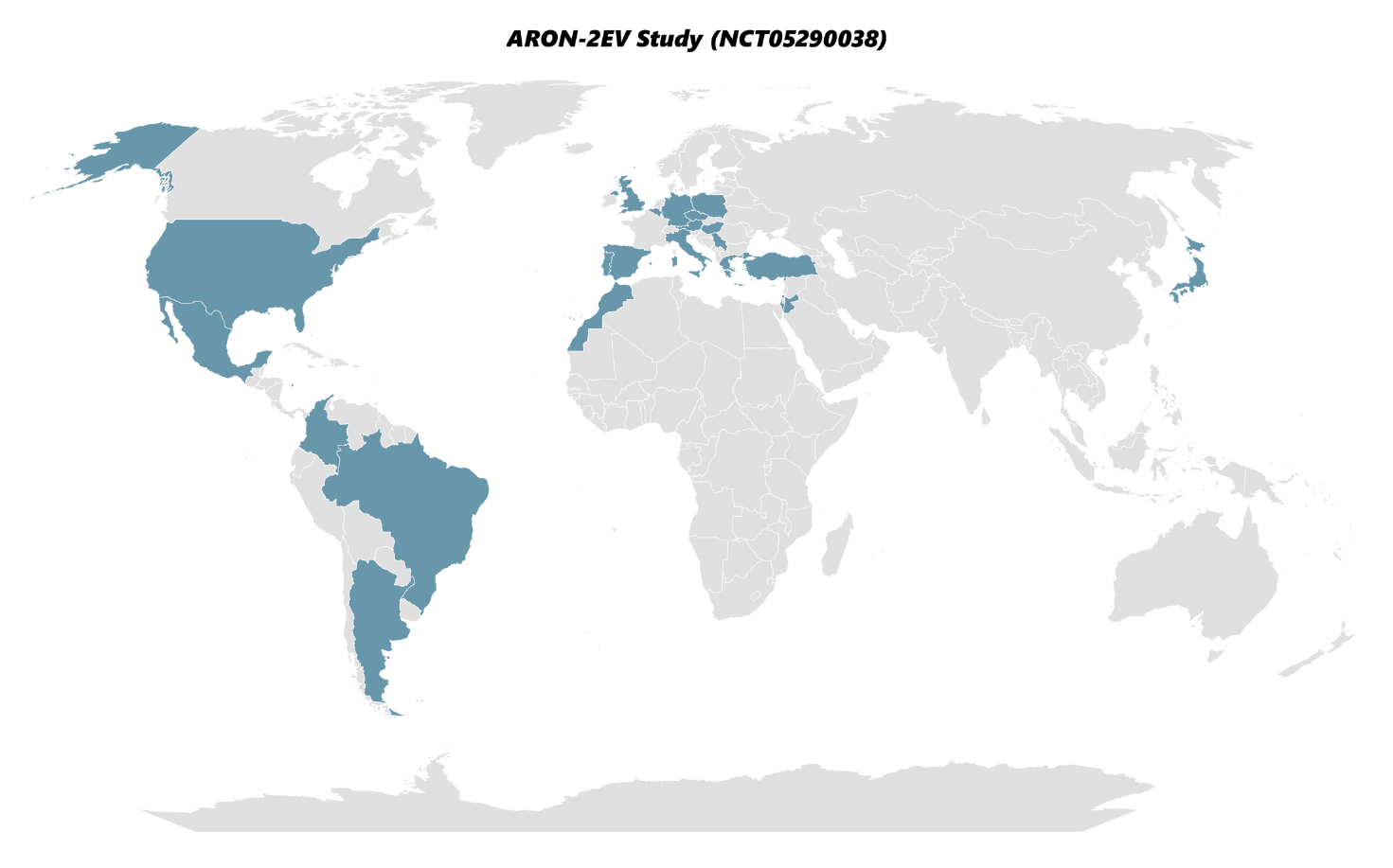


**Figure S2.** Selection process from the ARON-2EV dataset.

**
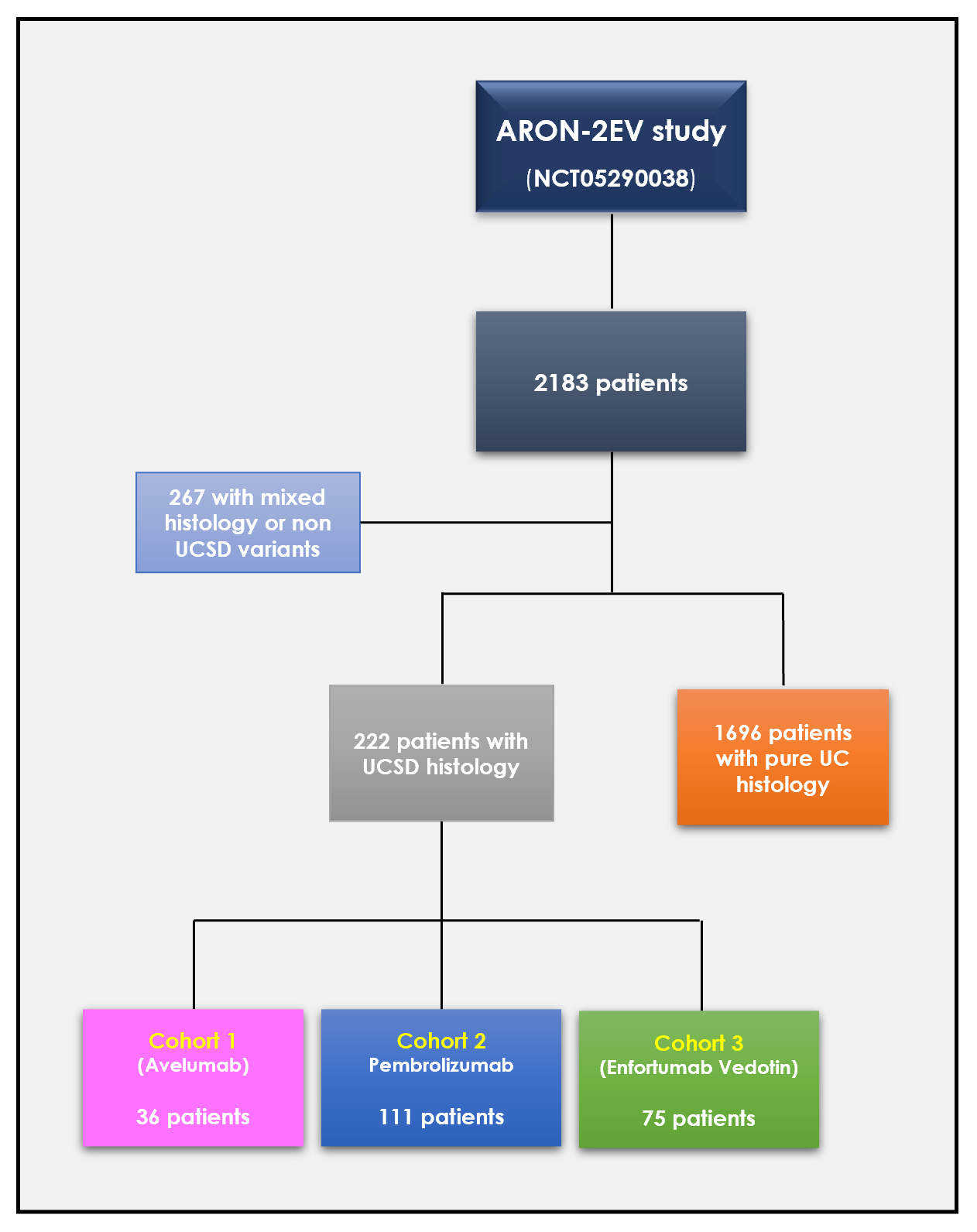
**
